# Telemedicine Training in Undergraduate Medical Education: Mixed-Methods Review

**DOI:** 10.2196/12515

**Published:** 2019-04-08

**Authors:** Shayan Waseh, Adam P Dicker

**Affiliations:** 1 Sidney Kimmel Medical College Thomas Jefferson University Philadelphia, PA United States; 2 Department of Radiation Oncology Sidney Kimmel Cancer Center Thomas Jefferson University Philadelphia, PA United States; 3 Jefferson Institute for Digital Health Thomas Jefferson University Philadelphia, PA United States

**Keywords:** telemedicine, education, medical, undergraduate, schools

## Abstract

**Background:**

Telemedicine has grown exponentially in the United States over the past few decades, and contemporary trends in the health care environment are serving to fuel this growth into the future. Therefore, medical schools are learning to incorporate telemedicine competencies into the undergraduate medical education of future physicians so that they can more effectively leverage telemedicine technologies for improving the quality of care, increasing patient access, and reducing health care expense. This review articulates the efforts of allopathic-degree-granting medical schools in the United States to characterize and systematize the learnings that have been generated thus far in the domain of telemedicine training in undergraduate medical education.

**Objective:**

The aim of this review was to collect and outline the current experiences and learnings that have been generated as medical schools have sought to implement telemedicine capacity-building into undergraduate medical education.

**Methods:**

We performed a mixed-methods review, starting with a literature review via Scopus, tracking with Excel, and an email outreach effort utilizing telemedicine curriculum data gathered by the Liaison Committee on Medical Education. This outreach included 70 institutions and yielded 7 interviews, 4 peer-reviewed research papers, 6 online documents, and 3 completed survey responses.

**Results:**

There is an emerging, rich international body of learning being generated in the field of telemedicine training in undergraduate medical education. The integration of telemedicine-based lessons, ethics case-studies, clinical rotations, and even teleassessments are being found to offer great value for medical schools and their students. Most medical students find such training to be a valuable component of their preclinical and clinical education for a variety of reasons, which include fostering greater familiarity with telemedicine and increased comfort with applying telemedical approaches in their future careers.

**Conclusions:**

These competencies are increasingly important in tackling the challenges facing health care in the 21st century, and further implementation of telemedicine curricula into undergraduate medical education is highly merited.

## Introduction

### The Context of Telemedicine in the United States

Telemedicine has grown exponentially over the past few decades in the United States. It is currently utilized by a majority of health care institutions and its market is expected to reach over US $40 billion in 2018 [1,2]. The current health care landscape in the United States likewise presents ideal conditions to accelerate this growth. A national physician workforce shortage, geographic maldistributions of primary care, and specialist physicians as well as an untenably high national health care expenditure, all serve to incentivize the telemedicine enterprise.

Telemedicine is an expansive field, distinct from but overlapping with telehealth, electronic health, and mobile health. One of the foundational questions preceding the development of any curricula is where to draw the lines in defining telemedicine. Sood et al found that telemedicine was the most fundamentally defined as the provision of health care services over a spatial distance through the use of telecommunication technology with the aim of providing benefit to a patient or population [3]. These benefits include the key aspects of the Triple Aim, such as the improvement of access to health care services, the reduction of health care costs for patients and for society, and the provision of more convenient and higher quality health care [4].

These benefits are rapidly becoming realized as more hospital systems, large employers and health insurance companies, individual states, and the federal government itself are increasingly turning toward telemedicine for health care solutions [1,5,6]. Implementation of a telemedicine visit program at a single rural Veterans Affairs hospital found reductions of over 820,000 miles of travel time for 1859 patients over the course of 9 years [7]. Ample evidence has also been generated supporting the influence that telemedicine exerts in improving health care outcomes in an array of different settings and conditions [8].

Underlying the unfoldment of telemedicine in the United States has been the concomitant emergence of enabling societal and cultural trends. With 84% of US adults reporting that they use the internet and 92% of US adults reporting that they own a cellphone, American society is more technologically equipped than ever before [9]. A willingness to rely on the utilization of such technology in answering health questions is increasingly apparent, with 70% of US adults reporting that their first source of medical information is searching the internet. These trends signify a ripening opportunity for telemedicine to fulfill the health care needs of an increasingly digitally enabled society that is willing and able to utilize modern technology.

Despite this reality, several barriers still remain in the widespread uptake of telemedicine as a health care delivery paradigm as common as traditional medical care. A Market Innovation Center Consumer Choice Survey characterized a number of consumer concerns regarding telemedicine utilization. Primary among these was the doubt regarding the quality of care delivered through telemedicine; other major concerns included the security of health information in the digital space, as well as the potential lack of personal connection with health care providers over telemedicine visits [10].

### The Role of Undergraduate Medical Education in Telemedicine

Training physicians to deliver high-quality, secure, and personable health care through telemedicine can serve to alleviate these concerns and promote the population-wide adoption of telemedicine. In fact, medical students who interact with telemedicine during their undergraduate medical training find that it contributes to the development of core competencies in patient care, medical knowledge, and practice-based learning; interestingly, these benefits tended to be stronger when telemedicine exposure occurs during undergraduate medical education as compared with during graduate medical education [11].

Finally, a number of concerns that hinder the adoption of telemedicine at the provider level, as well as system-wide level, have become increasingly apparent. These include concerns regarding legal and liability uncertainties, licensure requirements, and nascent reimbursement mechanisms [12]. Although these issues are becoming incrementally resolved at a governmental- and structural-level, undergraduate medical education can serve to equip future physicians with a more comprehensive understanding of the telemedicine space in their locality. Although the many uncertainties within the telemedicine field will take time to be delineated, effective and evolving telemedicine curricula can go a long way in encouraging future physicians to interact with telemedicine.

As telemedicine becomes more ubiquitous in our nation’s health care delivery system, it is imperative that modern physicians are trained to leverage such technology effectively. In this regard, undergraduate medical education represents an invaluable window of opportunity for building these capacities in future physicians. The American Medical Association (AMA) has similarly articulated the need for telemedicine training for medical students and residents and has subsequently encouraged its adoption by medical schools and other institutions [13].

The Liaison Committee on Medical Education’s (LCME) Annual Medical School Questionnaire from 2015 to 2016 shows that over a quarter of the nation’s allopathic degree-granting medical schools have implemented telemedicine training components into the preclinical phase of their curriculum and nearly half have implemented it into the clerkship phase [14,15]. The learning being generated by these institutions is encouraging and warrants deeper investment in preparing future physicians to be empowered utilizers of telemedicine technology.

The aim of this review was to characterize, both qualitatively and quantitatively, the diverse approaches being undertaken by medical schools and other health care institutions to implement telemedicine training into undergraduate medical education, and to allow for a better understanding of the current state of telemedicine capacity-building in undergraduate medical education in the United States. This will allow medical schools and other stakeholders to further develop their telemedicine capacity-building curricula in the most effective, systematic, and evidence-based way possible.

## Methods

### Literature Review

A literature review was carried out on Scopus using the terms and Boolean operators *telemedicine* AND *medical student* OR *undergraduate medical education* OR *medical school* to yield a total of 274 journal articles. There were 2 additional peer-reviewed journal articles in the Jefferson Digital Commons, which covered telemedicine education programs that were included. Of these 276 articles, 107 were excluded as they were either older than 10 years or did not cover the inclusion criteria of examining the implementation or evaluation of a telemedicine-related curriculum or program into undergraduate medical education, either in the preclinical or the clinical years of medical schooling. The texts of the remaining 169 journal articles were read to determine if they met the inclusion criteria; of these, 9 met the inclusion criteria and were included in the literature review. A similar search was carried out on Google Scholar but did not yield any additional journal articles relevant to the study.

The principal purpose for the literature review was to extract the components of the telemedicine training curriculum at each institution rather than to evaluate study design. Therefore, no formal quality evaluation of journal articles was carried out. Rather, each journal article was dissected to determine the features of the telemedicine training component being described, when in the curriculum it was included, and how it was implemented.

### Online Search and Survey

In addition, the LCME data from the AMA, which included statistics regarding telemedicine curricula implementation by US allopathic-degree-granting medical schools, were obtained. Using this information, all such schools marked as having some form of telemedicine curriculum were researched using a Web search for *telemedicine*, *telehealth*, *medical school*, *medical student*, and *medical education* to look for publicly available information regarding the telemedicine training within their curriculum. Any publicly available documents were downloaded and analyzed to determine the features of the telemedicine training component being described, when in the curriculum it was included, and how it was implemented.

In addition, for each medical school reporting to the AMA to have a telemedicine component within their curriculum, an appropriate contact was identified and contacted via an email explaining the research project, requesting a conversation to learn more about the institution’s telemedicine curriculum, and including a survey link for those that were unable to communicate via phone. This contact was the administrative or faculty member listed as being in charge of telemedicine within a medical school or associated health care system. When there was no such person, the Associate Dean or Deans of Curriculum were identified and contacted. The Checklist for Reporting Results of Internet E-Surveys (CHERRIES) for the distributed survey is included as Multimedia Appendix 1.

### Synthesis of Findings

Overall, through the online search for publicly available documents and the cross-sectional survey of faculty members responsible for telemedicine and/or medical school curriculum, 70 institutions were contacted and researched, yielding 7 interviews, 4 peer-reviewed research papers, 6 online documents, and 3 completed survey responses. These sources of information were primarily analyzed for the features of the telemedicine training component at each medical school, when in the curriculum such training was included, and how it was implemented. During the 7 interviews, additional questions were asked, which allowed stakeholders to share what they considered accelerators and barriers to the implementation of telemedicine in undergraduate medical education.

The information collected from the initial literature review, qualitative research, and quantitative information gathering were then synthesized into a congruent and comprehensive review of the current trends in telemedicine competency development in the domain of undergraduate medical education.

## Results

### Telemedicine in the Preclinical Phase

The preclinical years of undergraduate medical education represent an important window of opportunity for telemedicine training and exposure. At present, an array of medical schools across the country are generating learning regarding the implementation of telemedicine training into the preclinical years of undergraduate medical education.

**Figure 1:**
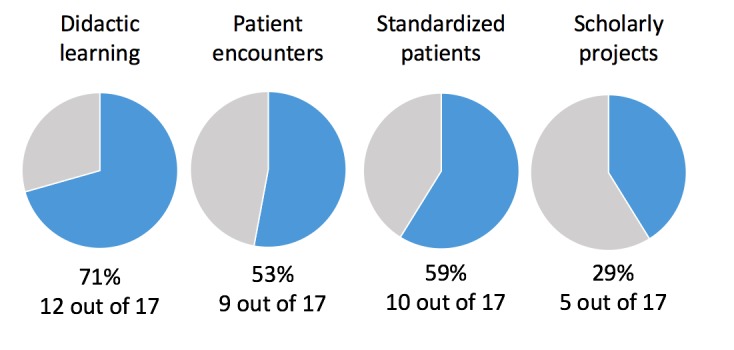
The format and auxiliary objectives of telemedicine curricula in US MD medical schools.

Twelve out of seventeen sampled medical schools with telemedicine curricula (71%) have implemented some form of didactic learning (Figure 1). In addition, 9 out of 17 (53%) and 10 out of 17 (59%) sampled medical schools utilize patient encounters or standardized patient encounters, respectively, to develop telemedicine competencies in medical students.

In addition, 5 out of 17 of sampled medical schools (29%) have incorporated telemedicine exposure into scholarly projects that medical students choose to pursue within a structured, but independent, framework.

The nature of telemedicine curricula has also been shown to lend itself to multipurpose implementation. Seven out of seventeen of sampled medical schools with telemedicine curricula (40%) combined telemedicine competencies with some form of interprofessional training and exposure (Figure 1).

In addition, a wide array of medical schools is finding that telemedicine training is a natural vehicle for exposing students to the considerations and concepts behind providing health care in rural settings. This is especially true of medical schools that have a vested interest in rural care, particularly those serving medical students in rural communities. In this regard, over half of sampled medical schools combined telemedicine competencies with rural medicine in some form.

**Figure 2:**
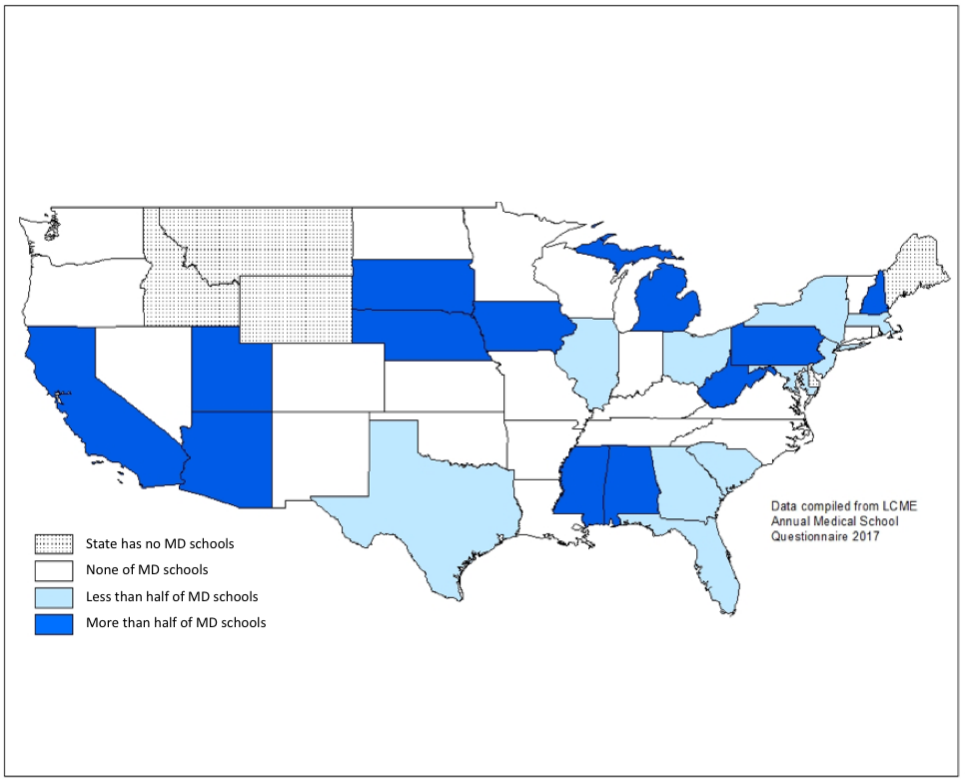
Proportion of US MD Medical Schools with a preclinical telemedicine curriculum by state. LCME: Liaison Committee on Medical Education; MD: Doctor of Medicine.

### Telemedicine in the Clerkship Phase

The clerkship phase of undergraduate medical education is where medical schools have, for the most part, invested the greatest amount of energy into telemedicine training for medical students. This is a result of the increasing presence that telemedicine has in the practice of clinical medicine itself; formalizing the telemedicine exposure experienced during clinical rotations is a natural process, particularly in fields where telemedicine is more commonly used such as psychiatry, neurology, dermatology, and radiology and in geographic areas where telemedicine is increasingly relied upon, such as the Midwest. It is no wonder then that over 60 allopathic medical schools in the United States provide some form of telemedicine experience in their clerkship offerings.

These clerkships range from rotations that simply afford exposure to the use of telemedicine in the course of everyday practice to distinct telemedicine electives that present a more focused opportunity for medical students to develop telemedicine competencies.

As telemedicine becomes increasingly implemented into the modern health care delivery paradigm, its presence in the wards during the clerkships of medical students will grow. Thereby, most medical students will gain at least basic exposure to telemedicine as a tool for providing health care. In addition, the emergence of telemedicine-focused electives provides an opportunity for motivated students to pursue further exposure to telemedicine and develop a stronger relevant skillset. In this way, quality telemedicine training is available to those that are interested but not mandated on others.

### Trends in Geographic Distribution

There is currently a large disparity between the implementation rates of telemedicine curricula among different states in the United States, particularly in regard to preclinical undergraduate medical education. There are a number of states that host a majority of schools that have implemented some form of telemedicine curriculum (Figure 2). Notable among these states are California, Pennsylvania, and Michigan, which together are home to over 1 out of every 7 medical schools in the country. There are however a number of large states where schools that have implemented telemedicine curricula in preclinical education are in the minority, including New York and Texas. Even more striking are the large swathes of the country where no medical schools include telemedicine training in any form as part of their preclinical undergraduate medical education curriculum. This is particularly concerning as these regions are often those that stand the most to benefit from telemedicine because of the large number of rural communities, such as in states like North Dakota, Kansas, and Oklahoma.

**Figure 3:**
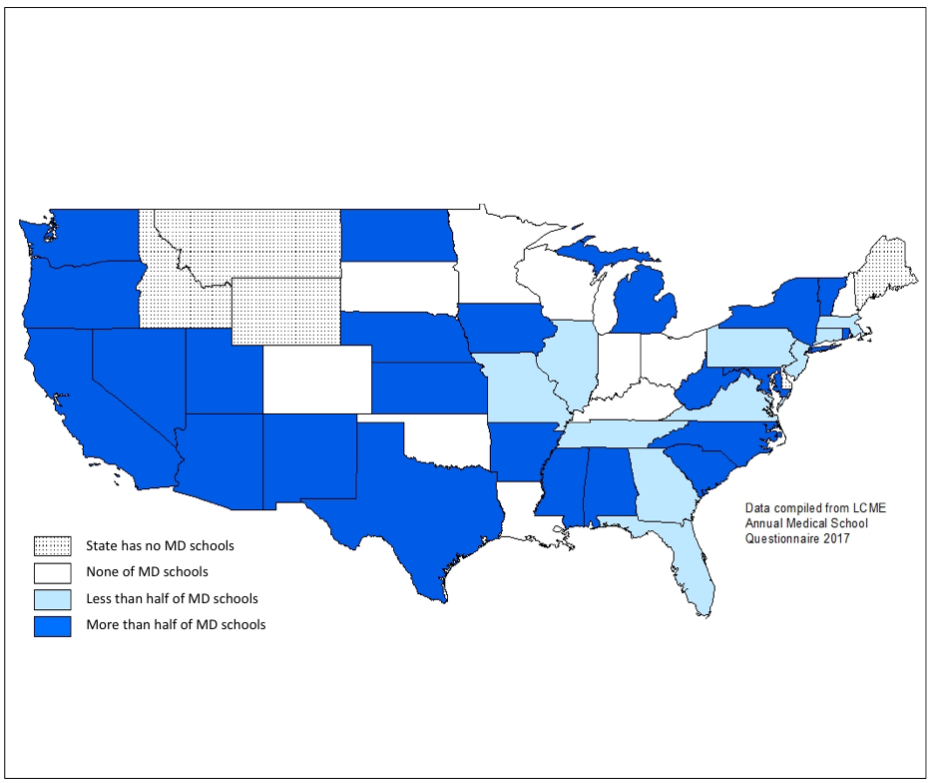
Proportion of US MD Medical Schools with a clinical telemedicine curriculum by state. LCME: Liaison Committee on Medical Education; MD: Doctor of Medicine.

There is a marked increase in the number and distribution of medical schools in the United States when considering schools that have implemented some form of telemedicine exposure or clerkship during the clinical years of their undergraduate medical education curriculum (Figure 3). This is often because of the natural exposure that students get to telemedicine when operating in settings where it is more commonly found, either with telemetry, remote specialist consults, and rural care. Most of the West Coast demonstrates high levels of telemedicine experience implementation in the clinical curriculum, as well as the rest of the country. There are however a number of states where these schools are the minority, including Florida and Pennsylvania, and there are still states that lack any schools with telemedicine exposure implemented into their curriculum, such as Minnesota, Wisconsin, and Oklahoma.

Overall, these findings characterize and illuminate the need that still exists within undergraduate medical education throughout the nation to incorporate telemedicine competencies and exposure into established medical school curricula. In fact, such efforts would be complimentary to the current work that medical schools, professional organizations, and local, state, and federal governments are already carrying out to improve the delivery of health care to rural and otherwise underserved populations.

### Contextual Forces in the Telemedicine Education Space

The importance of telemedicine training in the domain of undergraduate medical education has been clearly articulated by the AMA with the announcement of policy encouraging the adoption of telemedicine curricula by medical schools throughout the United States. However, as these telemedicine competencies are incorporated by more medical schools, attention should be given to the systematic implementation of programs and the scientific evaluation and dissemination of generated learning.

**Figure 4:**
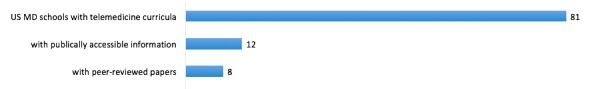
Availability of information regarding telemedicine curricula in undergraduate medical education in the United States. MD: Doctor of Medicine.

Transparency in the efforts of medical schools to incorporate telemedicine training into their curricula and discourse involving best practices needs to be fostered. At present, only 12 allopathic medical schools with telemedicine components in their preclinical or clerkship curriculum offer publicly available information regarding the format and content of such components (Figure 4).

In addition, an even smaller proportion of these medical schools have studied and published the impact of such training on medical students. This paucity of literature is a hindrance to the identification and dissemination of best practice approaches to telemedicine training in undergraduate medical education. A renewed commitment to the systematization and dissemination of knowledge is particularly justified by the nascent nature of telemedicine education in most parts of the United States; all medical schools will need to evolve their telemedicine competency components as the telemedicine landscape rapidly advances in coming years, and so all medical schools can benefit from open and knowledge-rich channels of communication.

Another trend influencing the telemedicine exposure that medical students receive during their undergraduate medical education is the emergence of strong telemedicine institutions and regional telemedicine networks. These organizations, often named telemedicine or telehealth centers, telemedicine programs, or telemedicine projects, confer upon medical students at associated institutions the ability to witness the vibrancy and scope of the telemedicine enterprise.

Project ECHO, for example, which was launched in New Mexico to allow specialists to assist primary care physicians through telemedicine, affords medical students a valuable opportunity to glimpse the capabilities of telemedicine to contribute to patient care at a population-wide level. Likewise, the Arizona Telemedicine Program serves as a strong regional resource for supporting telemedicine development and education. As more telemedicine programs at health care institutions develop, these centers for learning opportunities can be expected to play an even greater role in telemedicine training in undergraduate medical education.

## Discussion

### The Current Landscape of Telemedicine Training

The current state of telemedicine training in undergraduate education is that of budding growth and promising development; that more than a quarter of medical schools have preclinical telemedicine training in one or more diverse ways, and that almost half of medical schools have found organic ways to incorporate telemedicine exposure into students’ clerkship curriculum is promising. The geographic disparities that exist in the implementation of telemedicine training and the relationship between telemedicine exposure and the development of regional telemedicine institutes will be an important area of focus in the coming years.

Medical students have been shown to graduate feeling unprepared to utilize telemedicine effectively and feeling uninformed about the laws governing telemedicine use [16]. At the same time, there is a growing appreciation among medical students that such training would be both relevant and important for their future work [17]. Therefore, the acceleration of the implementation of telemedicine training into the undergraduate medical education curriculum in the United States is of vital importance. This is being accomplished through a diversity of institution-dependent avenues, including didactic learning, real patient and standardized patient encounters that develop telemedicine competencies, and scholarly projects that allow deeper insights into telemedicine technology.

The University of Maryland, for example, covers basic telemedicine concepts during lecture time, whereas the University of Nebraska has incorporated telemedicine into its doctoring thread. The Oregon Health and Sciences University has woven telemedicine into objective structured clinical examinations, allowing students to practice clinical skills using telemedicine technology while receiving formative feedback in a way that is financially feasible for the medical school and well-liked by students [18].

These all signify diverse approaches to fulfill the need for greater telemedicine exposure in undergraduate medical education. At the same time however, it is increasingly important that medical schools with telemedicine competencies are including them in their curriculum in a meaningful way. One of the greatest concerns among surveyed stakeholders was the meaningful use of telemedicine training and ensuring that telemedicine’s inclusion in the undergraduate medical education curriculum is more than cursory.

### The Future of Telemedicine Education

To accomplish this, telemedicine training in undergraduate medical education should move beyond the simple exposure of medical students to telemedicine technology and seek to augment such exposure with at least basic understanding of the complex governmental, socioeconomic, and cultural principles involved. This is especially important in light of the rapid pace of technological innovation in the telemedicine space; future physicians must not only be trained to use telemedicine but also to do so professionally, safely, and in an evidence-based manner [19].

The likely answer to this concern is already being explored by a multitude of medical schools that are finding ways to combine and consolidate different curricular aims into multifaceted educational components. By combining telemedicine training with existing competencies such as rural care exposure and interprofessional training, medical schools are able to expose future physicians to telemedicine without significant additional burden. Rather than struggling to fit telemedicine into an already overflowing curriculum, medical schools are most successfully able to include telemedicine competencies when they build them into existing components of the curriculum.

The Cleveland Clinic, for example, has incorporated telemedicine into an ethics curriculum, allowing a panel of second-year medical students to interview dialysis patients over a live video stream to learn about professionalism, patient experiences, and health care ethics [20]. At the University of Arizona (Tucson) and the University of North Dakota, telemedicine is being used to foster interprofessional training and collaboration among students from different health professions [21].

Telemedicine in the clerkship phase of the undergraduate medical education curriculum is another immensely important area of focus and is where the deepest level of development has occurred in regard to inclusion of telemedicine training in meaningful ways. Medical students that participate in telemedicine electives view telemedicine as an important educational tool and highly rate the ability of telemedicine to contribute to their medical knowledge, patient care skills, and system-based practice [11,22].

At the University of New Mexico, medical students are exposed to telemedicine as they rotate through a variety of clerkships, and students who show an interest are able to develop research projects and community interventions that utilize telemedicine. The scope of these projects has even included telemedicine in a global health context with students working abroad. As such, telemedicine training during the clerkship phase of medical education also represents a valuable opportunity for student learning to intertwine with genuine contributions to worldwide health [23].

Medical schools such as Thomas Jefferson University, the University of Texas Medical Branch (Galveston), the University of Texas (Houston), and Southern Illinois University have all implemented distinct telemedicine clerkships. At Thomas Jefferson University, third- and fourth-year medical students can participate in an elective where they aid patients and the medical team in carrying out virtual rounds, which allow patient families to participate during rounds through telemedicine [24].

At the University of Texas Medical Branch (Galveston), medical students learn about the field of telemedicine through study and exposure in multiple different practice settings. All participating students found that the experience proved helpful in focusing their future career goals and shared that they would recommend such an elective to fellow students [25].

### The Barriers to Overcome for Widespread Adoption of Telemedicine Training

Although there is an immense amount of knowledge to be gained from exploring the current state of telemedicine in undergraduate medical education in the United States, there are important barriers and limitations to consider. At many institutions, telemedicine exposure exists within the curriculum only implicitly, which prevents quantitative analysis, whereas at other institutions, telemedicine competencies may be formally included in the curriculum but not actually implemented in practice. In addition, as the number of medical schools in the United States is large, the low sample size of 17 is enough to give a general snapshot of the state of telemedicine education but not an exhaustive understanding. Indeed, there is a plethora of knowledge to be gained from further research and analysis regarding telemedicine in undergraduate medical education.

Future areas of research will undoubtedly involve comparing the efficacy of existing telemedicine training components and studying the most effective way to implement telemedicine into institutions with no telemedicine training and to evolve the current telemedicine trainings that medical schools are carrying out. Importantly, the state and regional geographic disparities in the rate of inclusion of telemedicine training into the undergraduate preclinical and clerkship curriculum are an important phenomenon that surely influence the education of the future generation of physicians and must be better understood.

At the same time, it is important to understand the global context within which the telemedicine training in the United States exists. Compared with countries such as Australia, which have relied heavily on telemedicine because of geographic limitations, the United States has much development to accomplish. However, compared with other countries such as France, the spread and reach of telemedicine training in undergraduate medical education is advance [26]. There therefore seems to be an important correlation between the state of telemedicine itself within a country, and the development of the educational system necessarily to effectively utilize telemedicine.

As telemedicine has become an increasingly important presence in the health care system of the United States, its inclusion into the training of future physicians has likewise become increasingly necessary and important. The diverse approaches being undertaken by medical schools in developing telemedicine competencies in medical students is a promising sign of accelerating growth in this domain, but future effort is needed on the part of institutions to make such training meaningful and comprehensive. Further research into telemedicine training in undergraduate medical education will be an important part of the process and will be an area of need in coming years.

## Appendix

Multimedia Appendix 1 The Checklist for Reporting Results of Internet E-Surveys (CHERRIES).

## Abbreviations

AMA: American Medical Association
LCME: Liaison Committee on Medical Education
