# Supplementary material for: Telemedicine Training in Undergraduate Medical Education: Mixed-Methods Review
Source: JMIR Med Educ. 2019 Apr 8;5(1):e12515. doi: 10.2196/12515 (PMC6475822; doi:10.2196/12515)
Supplement: Multimedia Appendix 1 [file mededu_v5i1e12515_app1.pdf]

Item Category	Explanation
Design	The e-surveys of this study were aimed to collect information from the medical school administrator responsible for telemedicine at medical schools with telemedicine training in their undergraduate medical education
IRB	IRB approval was not necessary
Development and Pre-testing	The survey was developed using SurveyGizmo based on the themes identified in the literature review. It was pre-tested for functionality by the research team.
Recruitment process	The data was collected using a linked survey.<br><br>Initial contact with participants was made through email to each participant's institutional email address. An email was sent explaining the research project and requesting a phone call to interview them or for them to complete the survey form. The email included a link to the survey form.<br><br>The survey was not otherwise advertised.
Survey administration	The survey was sent through email and data was entered automatically when participants responded to the survey questions.<br><br>The survey was voluntary and no incentives were offered.<br><br>The survey occurred in 2017.<br><br>There was no randomization of questions or adaptive questions.<br><br>There were 4 questions in the survey.<br><br>The survey did not need to be complete for the participant to submit their responses. Participants were able to review their responses before submitting.
Response rates	The response rate was 3 survey responses out of 70 individuals contacted (4%). However, an additional 7 interviews occurred

	outside of the surveys as a result of the email contact (10%).
Preventing multiple entries	Participants entered the institution with which they were affiliated to ensure that each institution was not represented in the survey responses more than once.
Analysis	Survey responses were qualitatively analyzed to determine where and how in the undergraduate medical education curriculum telemedicine training was included at their institution.
